# Comparison of OneChoice AI-Based Clinical Decision Support Recommendations with Infectious Disease Specialists and Non-Specialists for Empirical Urinary Tract Infection Therapy in Lima, Peru

**DOI:** 10.3390/diagnostics16172708

**Published:** 2026-08-25

**Authors:** Juan Carlos Gómez de la Torre, Ari Frenkel, Carlos Chavez-Lencinas, Alicia Rendon, Max Fabian, José Alonso Cáceres-DelAguila, Diana Minchon-Vizconde, Miguel Hueda-Zavaleta

**Affiliations:** 1 Clinical Laboratory Roe, Lima 15076, Peru; jcaceres@labroe.com; 2 Arkstone Medical Solutions, Boca Raton, FL 33428, USA; afrenkel@arkstonemedical.com (A.F.); clencinas@arkstonemedical.com (C.C.-L.); arendon@arkstonemedical.com (A.R.); max.fabian@labroe.com (M.F.); 3 Instituto de Investigaciones en Ciencias Biomedicas, Universidad Ricardo Palma, Lima 15039, Peru; 4 Facultad de Medicina, Universidad Ricardo Palma, Lima 15039, Peru; 5 Hospital Nacional Edgardo Rebagliati Martins, Lima 15073, Peru; 6 Facultad de Medicina, Universidad Nacional Mayor de San Marcos, Lima 15072, Peru; 7 Facultad de Ciencias de la Salud, Universidad Privada de Tacna, Tacna 23001, Peru; diana.vizconde@arkstonemedical.com

**Keywords:** urinary tract infection, antimicrobial stewardship, clinical decision support system, machine learning, physician decision making

## Abstract

**Background:** Antimicrobial resistance complicates the selection of appropriate regimens for urinary tract infections (UTIs), even when susceptibility data are available, particularly where infectious disease (ID) expertise is scarce. Machine learning clinical decision support systems (CDSS) may support prescribing, but evidence from Latin America is limited. The goal of this study was to evaluate the concordance between antimicrobial regimens selected by physicians and those recommended by a machine-learning-with-human-in-the-loop (ML-HITL) CDSS (OneChoice^®^), and to assess CDSS appropriateness against an independent, blinded expert reference standard. **Methods:** In this cross-sectional, survey-based concordance study conducted in Lima, Peru, 194 verified physicians contributed 224 eligible evaluations across 42 real UTI case codes with complete culture and antimicrobial susceptibility data. Of the 224 evaluations, 70 were contributed by infectious disease specialists and 154 by non-ID physicians. Participants selected OneChoice^®^ and alternative antimicrobial regimens. Responses were compared with CDSS recommendations under three concordance definitions. Discordances were adjudicated by an external panel blinded to the source of the recommendation. Non-independence was addressed using cluster-robust methods. **Results:** First-choice, alternative, and general concordance were 50.9%, 40.6%, and 62.5%, respectively. ID specialists showed higher concordance than non-ID physicians (65.7% vs. 44.2%; 51.4% vs. 35.7%; 72.9% vs. 57.8%; all *p* ≤ 0.034). ID specialty was independently associated with concordance (adjusted OR 2.19–2.68; *p* ≤ 0.016). Among discordant evaluations, the external panel judged the CDSS recommendation to be preferable in 90.5–93.2% of cases. Physician–CDSS concordance was moderate and higher among ID specialists. The external adjudication findings indicate that the CDSS recommendations were frequently aligned with expert assessment when physician and CDSS recommendations differed; however, the study did not evaluate comparative clinical effectiveness or patient outcomes. **Conclusions:** Physician–CDSS concordance was moderate and higher among ID specialists, yet discordances overwhelmingly favored the CDSS on independent adjudication. These findings suggest the CDSS aligns with expert reasoning and may support antimicrobial selection in high-resistance settings.

## 1. Introduction

Urinary tract infections (UTIs) rank among the most common bacterial infections worldwide and place a sustained burden on health systems. The Global Burden of Disease Study estimated 404.6 million UTI cases and 236,790 attributable deaths in 2019, with the age-standardized mortality rate rising from 2.77 to 3.13 per 100,000 between 1990 and 2019 [1]. More recent GBD modeling reported a 66.5% increase in cases, reaching 4.49 billion in 2021, with *Escherichia coli* identified as the pathogen responsible for the highest AMR-related UTI mortality [2]. The lifetime risk of UTI is estimated at 93.7% and reaches 96.1% in females [3]. In ambulatory care alone, US national surveys document an annualized prevalence of 1511 cases per 100,000 persons—close to 10 million encounters per year—with more than 55% of episodes occurring in adults aged 65 years or older [4]. In Latin America, a meta-analysis of 111,249 pregnant women estimated a prevalence of asymptomatic bacteriuria, lower UTI, and pyelonephritis of 18.5%, 7.5%, and 2.3%, respectively, with *E. coli* accounting for approximately 70% of isolates [5].

Antimicrobial resistance (AMR) is amplifying this burden. In 2021, bacterial AMR was associated with 4.71 million deaths globally and was directly attributable to 1.14 million [6]; for UTIs specifically, an estimated 64.89 thousand deaths in 2019 were directly attributed to AMR [7]. Resistance among uropathogens is particularly concerning in Latin America. SENTRY-derived data documented community-acquired *E. coli* resistance of 40.4% to trimethoprim/sulfamethoxazole and 21.6% to ciprofloxacin [8], and a recent prospective cohort in low-resource Bolivia reported resistance rates of 63.8% and 56.4% to the same agents, with 26.7% of isolates carrying *bla*CTX-M genes [9]. In European emergency departments, the UTILY cohort showed that 35.9% of Enterobacterales causing UTI were non-susceptible to third-generation cephalosporins, with a 30-day mortality of 10.1% [10]. Extended-spectrum beta-lactamase (ESBL)-producing *E. coli* accounted for 38.97% of inpatient isolates in long-term hospital surveillance [11]. The WHO 2024 Bacterial Priority Pathogens List ranked carbapenem-resistant *Klebsiella pneumoniae* as the highest-priority pathogen (84%), reinforcing the urgency for stewardship-aligned empirical therapy [12].

Empirical decisions in UTI continue to rely on urine culture and phenotypic susceptibility testing, which remain the reference standard but require 24–72 h [13]. Real-world data show that this latency translates into substantial inappropriate prescribing: in a retrospective cohort of 120,519 English patients with uncomplicated UTI, 43.5% received guideline-discordant therapy [14]; and in a recent ED cohort, empirical regimens were inadequate in 63% of febrile UTIs caused by 3GC-resistant Enterobacterales [15].

Artificial intelligence (AI) and machine learning (ML) have emerged as decisive tools to bridge this gap. A recent meta-analysis showed that ML-based models outperformed traditional risk-scoring systems in sensitivity (1.93, 95% CI 0.48–3.39, *p* = 0.009) and negative predictive value (1.66, 95% CI 0.86–2.46, *p* < 0.001) for stewardship-related outcomes [16]. A systematic review reported that ML-driven clinical decision support systems (CDSS) reduced extended-spectrum antibiotic days of therapy by 17.4% in UTIs and by 28.4% in pneumonia [17]. ML applications dedicated to UTIs have shown areas under the ROC curve of 0.84 for fluoroquinolone resistance [18], 0.81 for ciprofloxacin in complicated UTI [19], and up to 0.96 in surveillance datasets [20]. A scoping review of 11 real-time CDSSs further described consistent reductions in broad-spectrum antibiotic use, mortality, and costs [21].

OneChoice^®^ (Arkstone Medical Solutions, New York, NY, USA), an ML-with-human-in-the-loop (ML-HITL) CDSS, was built specifically to translate phenotypic and molecular results into stewardship-aligned empirical regimens. Internal validation reported 100% accuracy in distinguishing trained from novel data, 84% agreement with clinical standards in minor discrepancies, and zero major discrepancies [22]. In bacteremia, our group has shown that OneChoice^®^ delivered 80% concordance between recommendations issued from molecular and phenotypic data, with molecular-driven recommendations available up to 29 h earlier than conventional outputs [23]. In a head-to-head survey of 366 case evaluations in Lima, the system achieved 96.1% concordance with physicians for any suggested treatment and 74.6% for the top recommendation (κ = 0.70), with infectious disease (ID) specialists reaching higher agreement (κ = 0.78) than non-ID physicians (κ = 0.61) [24]. A more recent comparison with general-purpose large language models confirmed that the ML-HITL approach achieved the highest concordance with an independent panel of ID experts across 88 positive urine culture cases [25].

Despite this evidence, no study has yet assessed the alignment between an AI-CDSS and physicians of multiple specialties for empirical UTI therapy in Latin America, where high resistance rates and uneven access to ID consultation make stewardship particularly challenging [26,27]. We therefore conducted a survey-based study in Lima, Peru, in which physicians from infectious diseases and other specialties responded to real UTI vignettes presented in randomized order. Our objective was to compare the recommendations issued by OneChoice^®^ with those of ID specialists and non-specialists for empirical UTI therapy, and to determine whether the system’s performance is consistent.

## 2. Materials and Methods

### 2.1. Study Design and Setting

An observational, cross-sectional, survey-based concordance study was conducted using retrospectively selected real urinary tract infection (UTI) cases. The study evaluated the concordance between antimicrobial treatment options selected by physicians from multiple specialties and the recommendations generated by a machine-learning-with-human-in-the-loop (ML-HITL) clinical decision support system (CDSS; OneChoice^®^, Arkstone Medical Solutions, NY, USA). The study was conducted in Lima, Peru. Clinical cases were derived from anonymized real-world cases studied at Laboratorios Roe between January and September 2024. Three investigators specializing in infectious and tropical diseases prepared the clinical vignettes using real clinical and microbiological data, including the corresponding positive urine culture and phenotypic antimicrobial susceptibility results. The same clinical and laboratory information was transmitted to the CDSS to generate the OneChoice^®^ recommendations. The cases were derived from diagnostic records processed at Laboratorios Roe rather than prospectively recruited from a single hospital ward, outpatient clinic, or private office. All patient information was anonymized before vignette preparation. Data extraction and preparation were performed between 15 June and 25 June 2025, and the physician survey was administered between 26 June and 15 July 2025.

The study and manuscript were reported in accordance with the Strengthening the Reporting of Observational Studies in Epidemiology (STROBE) guidance for cross-sectional studies; the completed checklist is provided as Supplementary S1.

### 2.2. Questionnaire Selection and Development

Eligible cases were required to contain sufficient clinical information, a positive urine culture, an antimicrobial susceptibility profile, and an isolate considered clinically consistent with the urinary syndrome rather than contamination. Cases with insufficient clinical information, absent susceptibility results, or isolates considered likely contaminants were excluded.

A total of 87 QR-linked codes were initially generated. Of these, 6 codes were used exclusively for technical testing, leaving 81 study case codes. All 81 study case codes were made available through the survey. The case codes represented anonymized real-world UTI cases containing the clinical and microbiological information used to construct the vignettes and to generate the corresponding OneChoice^®^ recommendations. The number of case codes represented in the final analytical dataset was determined subsequently during database analysis, after evaluation-level eligibility and data-quality exclusions. The 42 retained questionnaires included real UTI cases of varying clinical complexity, comprising both complicated and uncomplicated UTI, with uncomplicated cases being the most frequent. Complicated UTI was defined, following IDSA/EAU criteria, as infection associated with ≥1 of the following: male sex, pregnancy, urinary tract structural or functional abnormality, indwelling catheter, immunosuppression, or healthcare-associated acquisition. These criteria correspond to the definitions in force at the time of the study, prior to the most recent IDSA revisions.

Each questionnaire contained one clinical vignette based on real-world clinical and microbiological information. When available, it included clinical and epidemiological variables such as age, sex, symptoms, pregnancy status, comorbidities, allergies, previous antibiotic exposure, recent hospitalization, renal function, isolated pathogen, and antimicrobial susceptibility profile. Because the questionnaires were derived from routine clinical records, the amount and structure of clinical information varied across cases. Each questionnaire included a structured display or image of the identified pathogen and its antibiogram; therefore, the task evaluated antimicrobial treatment selection based on culture and susceptibility results rather than on empirical therapy.

Each questionnaire included two sequential questions. The first asked physicians to select the best first-choice antimicrobial regimen; the second asked them to select the best alternative regimen if the first option was unavailable. Both questions used the same clinical vignette and the same antibiogram.

For each questionnaire, the OneChoice^®^ generated a priori one first-choice and one alternative antimicrobial regimen based on the clinical information in the vignette, the antimicrobial susceptibility profile, and current therapeutic guidance. A physician’s response was considered concordant with the OneChoice^®^ only when the antimicrobial agent, dose, dosing interval, and treatment duration all matched the OneChoice^®^-generated option; responses with the correct agent but an incorrect dose, interval, or duration were not considered concordant. Example questionnaire in Supplementary S2.

Questionnaire allocation was generated automatically through a script hosted in a public GitHub (https://maxfabian16.github.io/survey-randomizer/; Accessed on 15 August 2026) repository. The script generated a public link via a QR code, and each QR scan randomly directed the participant to one of the study case codes. The QR link remained accessible throughout the survey period, and the same physician could access it more than once; therefore, some physicians completed more than one randomly allocated vignette. Allocation was not stratified and did not involve investigator-directed case assignment.

### 2.3. Participants and Selection Criteria

Physicians practicing principally in Lima, Peru, were invited to participate voluntarily through non-probabilistic convenience sampling. Recruitment was conducted through professional contacts, hospital networks, medical congresses, and electronic communication channels across public and private healthcare facilities. Physicians involved in clinical care and with routine exposure to UTI management were eligible; both infectious disease specialists and physicians from other specialties were included. During database analysis, individual evaluations were retained only when all required eligibility and analytical information was available, particularly a verifiable medical registration number. Evaluations lacking the required information were excluded independently of the respondent’s therapeutic choices, concordance outcomes, or medical specialty. No financial compensation or material incentive was provided.

The final analytical dataset comprised 224 physician–case evaluations, of which 70 were contributed by ID specialists and 154 by non-ID physicians. These evaluations were contributed by 194 unique physicians and involved 42 distinct clinical questionnaires. Questionnaires were randomly assigned from the pool of 42 eligible questionnaires. Consequently, the data had a cross-classified structure: a given questionnaire could be evaluated by more than one physician, and a given physician could evaluate more than one questionnaire; however, no physician evaluated the same questionnaire more than once. Repeated use of questionnaires therefore arose through random assignment rather than intentional oversampling.

### 2.4. AI-Enabled Clinical Decision Support System

The ML-HITL CDSS evaluated in this study was OneChoice^®^ (Arkstone Medical Solutions, NY, USA), a human-in-the-loop system whose internal and external validation has been previously published [22]. In routine laboratory practice, the system generates antimicrobial recommendations as a complementary interpretive tool alongside urine culture and susceptibility testing, providing a primary recommendation and alternative options that may be adjusted based on available microbiological information. For the selected questionnaires, OneChoice^®^ recommendations corresponded to the same period as the source urine cultures and were generated using the clinical and microbiological information available for each case, including urine culture results and antimicrobial susceptibility profiles.

### 2.5. Ethical Considerations

The study protocol was reviewed and approved by the Research Ethics Committee before implementation (FACSA-CEI/054-06-2026). Because the questionnaires were derived from anonymized, previously archived clinical and microbiological records, the committee waived the requirement for individual informed consent from source patients. All questionnaires were anonymized before administration, and no direct patient identifiers were included in the survey instrument or analytic database. Participating physicians provided electronic informed consent before completing the survey; participation was voluntary, no compensation was provided, and participants were offered access to the aggregate study results after study completion.

### 2.6. Data Preparation and Outcome Definitions

Survey responses were anonymized using numeric identifiers for physicians and questionnaires and consolidated in Microsoft Excel. During database analysis, evaluations lacking required eligibility or analytical information, particularly a verifiable medical registration number, were excluded. These exclusions were applied independently of the respondents’ therapeutic choices, concordance outcomes, or medical specialty. After these evaluation-level exclusions, 39 of the 81 study case codes had no eligible evaluations remaining. The final analytical database therefore contained 224 eligible physician–case evaluations contributed by 194 verified physicians across the remaining 42 case codes. Of the 194 physicians, 169 completed one vignette, 20 completed two vignettes, and 5 completed three vignettes. The final dataset had no missing values for the three concordance outcomes or for the covariates included in the multivariable models. All 224 retained evaluations were included in the complete-case analyses.

Following verification of eligibility, completeness, and uniqueness of each physician–questionnaire combination, the final analytical database contained exactly 224 physician–case evaluations contributed by 194 unique physicians across 42 clinical questionnaires. The final dataset had no missing values for the three concordance outcomes or for the covariates included in the multivariable models. All 224 retained evaluations were included in the complete-case analyses.

Three concordance outcomes were defined for each evaluation. First-choice concordance was defined as agreement between the participant’s first-choice regimen and the OneChoice^®^ first-choice recommendation. Alternative concordance was defined as agreement between the participant’s alternative regimen and the OneChoice^®^ alternative recommendation. General concordance was defined as agreement between either of the participant’s two regimens and any OneChoice^®^-proposed regimen (first-choice or alternative), capturing clinically acceptable agreement under a less restrictive criterion. Discordant evaluations were categorized by type of mismatch (incorrect antimicrobial choice, dose, dosing interval, or treatment duration; unnecessary broad-spectrum coverage; unnecessary carbapenem use; or combined errors).

Completeness was assessed for all outcome and model-covariate fields before analysis. No values were missing for the three concordance outcomes or for physician specialty, physician age, years of specialty experience, UTI classification, or causative organism. No imputation was performed, and complete-case analysis included all 224 retained evaluations.

### 2.7. Data Analysis

Descriptive statistics summarized case- and physician-level characteristics as *n* (%) or mean ± standard deviation (median, IQR for non-normally distributed variables); differences between ID and non-ID groups were tested with χ^2^ or Fisher’s exact test for categorical variables and with Student’s *t* or Mann–Whitney U test for continuous variables. Concordance proportions were reported with 95% Wilson confidence intervals under each adjudication method and for each of the three outcomes.

The unit of analysis was the individual physician–case evaluation. Because some physicians evaluated more than one questionnaire and individual questionnaires were evaluated by multiple physicians, the observations had a cross-classified correlation structure. Inferential comparisons between specialty groups and multivariable logistic regression models therefore used two-way cluster-robust covariance estimation, with clustering at both the physician and clinical-case levels and finite-sample corrections. This approach simultaneously accounted for within-physician correlation and for correlation among evaluations of the same clinical case. Wilson confidence intervals were retained as descriptive intervals for the unadjusted concordance proportions, whereas hypothesis tests and regression confidence intervals were based on the two-way cluster-robust covariance estimator.

For the subgroup analyses, we computed the difference in paired proportions Δ = (OneChoice^®^ vs. gold standard) − (participants vs. gold standard), with 95% confidence intervals, stratified by physician age, ID/non-ID status, years of specialty experience, UTI classification (uncomplicated/complicated), causative organism (*E. coli* versus non-*E. coli*), and resistance phenotype (susceptible, ESBL-positive, fluoroquinolone-resistant, multidrug-resistant MDR).

Independent predictors of physician concordance with OneChoice^®^ were examined using multivariable logistic regression fitted separately for the primary, alternative, and general concordance outcomes. The prespecified covariates included physician age, ID specialty, years of specialty experience, complicated UTI, and causative organism (*Escherichia coli* versus other pathogens). Because years of specialty experience was collected in five ordered ranges, category midpoints were used to represent this variable as a continuous measure. Results are reported as adjusted odds ratios with 95% confidence intervals and two-sided *p*-values based on two-way cluster-robust standard errors.

Three other multivariable logistic regression models were fitted separately for primary, alternative, and general concordance. The final predictor set was specified based on clinical relevance and model parsimony and included ID specialty, physician age, years of specialty experience, UTI classification, causative organism (*E. coli* versus other organisms), and years of specialty experience. The number of predictors was restricted to preserve an adequate events-per-predictor ratio. Multicollinearity was evaluated using variance inflation factors, with VIF values above 5 considered indicative of potentially important multicollinearity. Model convergence was verified, overall fit was evaluated using the likelihood-ratio and Hosmer–Lemeshow tests, and discrimination was summarized using the C-statistic. Brier scores were calculated as an overall measure of probabilistic prediction error. Adjusted odds ratios and 95% confidence intervals were estimated using two-way cluster-robust standard errors at the physician and clinical-case levels. Statistical significance was defined as *p* < 0.05. All analyses were conducted in Python 3.11 (pandas, numpy, scipy, statsmodels).

## 3. Results

**Figure 1:**
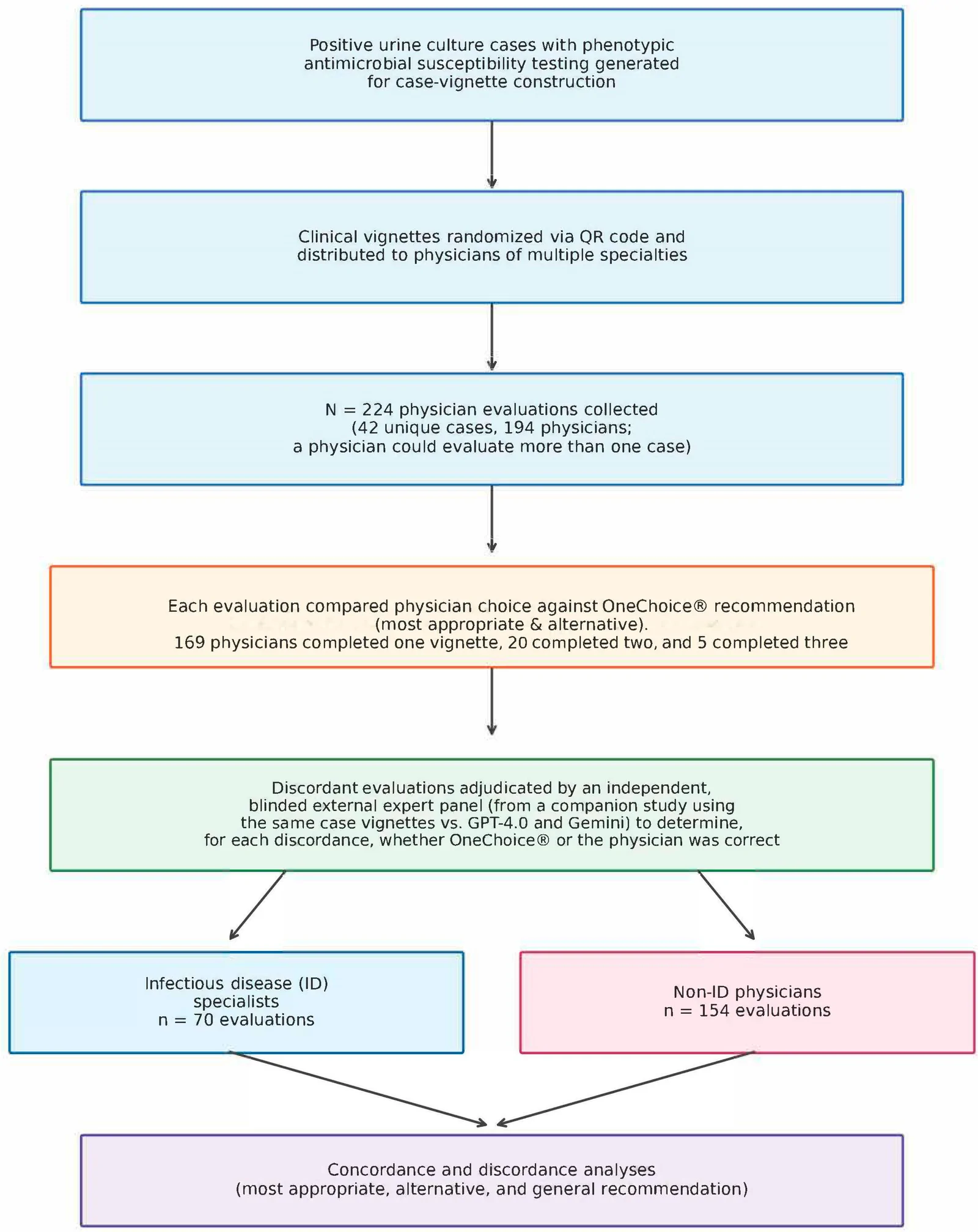
Study flow diagram.

A total of 87 QR-linked codes were initially generated, of which 6 were technical test codes, leaving 81 study case codes. All 81 study case codes were made available through the survey, with each QR access randomly directing the participant to a clinical case. During database analysis, evaluations lacking required eligibility or analytical information, particularly a verifiable medical registration number, were excluded independently of the respondents’ therapeutic choices, concordance outcomes, or medical specialty. Consequently, 39 of the 81 study case codes had no eligible evaluations remaining. The remaining 42 case codes generated 224 eligible physician–case evaluations from 194 verified physicians. Of these physicians, 169 (87.1%) completed one vignette, 20 (10.3%) completed two, and 5 (2.6%) completed three. Of the 224 evaluations, 70 (31.3%) were contributed by infectious disease specialists and 154 (68.8%) by non-ID physicians (Table 1 and Figure 1).

Baseline case characteristics were well balanced between the two groups. *Escherichia coli* was the most frequent causative organism (152/224, 67.9%), followed by *Klebsiella pneumoniae* (26/224, 11.6%); ESBL-positive, fluoroquinolone-resistant, and multidrug-resistant isolates accounted for 37.1%, 47.3%, and 39.3% of evaluations, respectively, with no significant differences by specialty (all *p* ≥ 0.64) except for a lower frequency of *Proteus mirabilis* cases among ID specialists (1/70 vs. 15/154; *p* = 0.050) (Table 1).

Overall, participants agreed with the OneChoice^®^ recommendation in 114/224 evaluations (50.9%, 95% CI 44.4–57.4) for the most appropriate antimicrobial regimen, in 91/224 (40.6%, 95% CI 34.4–47.2) for the alternative recommendation, and in 140/224 (62.5%, 95% CI 56.0–68.6) for general concordance (agreement with at least one of the two recommendations). Confidence intervals for all proportions were calculated using the Wilson score method, which is appropriate for proportions close to 0 or 1.

**Figure 2:**
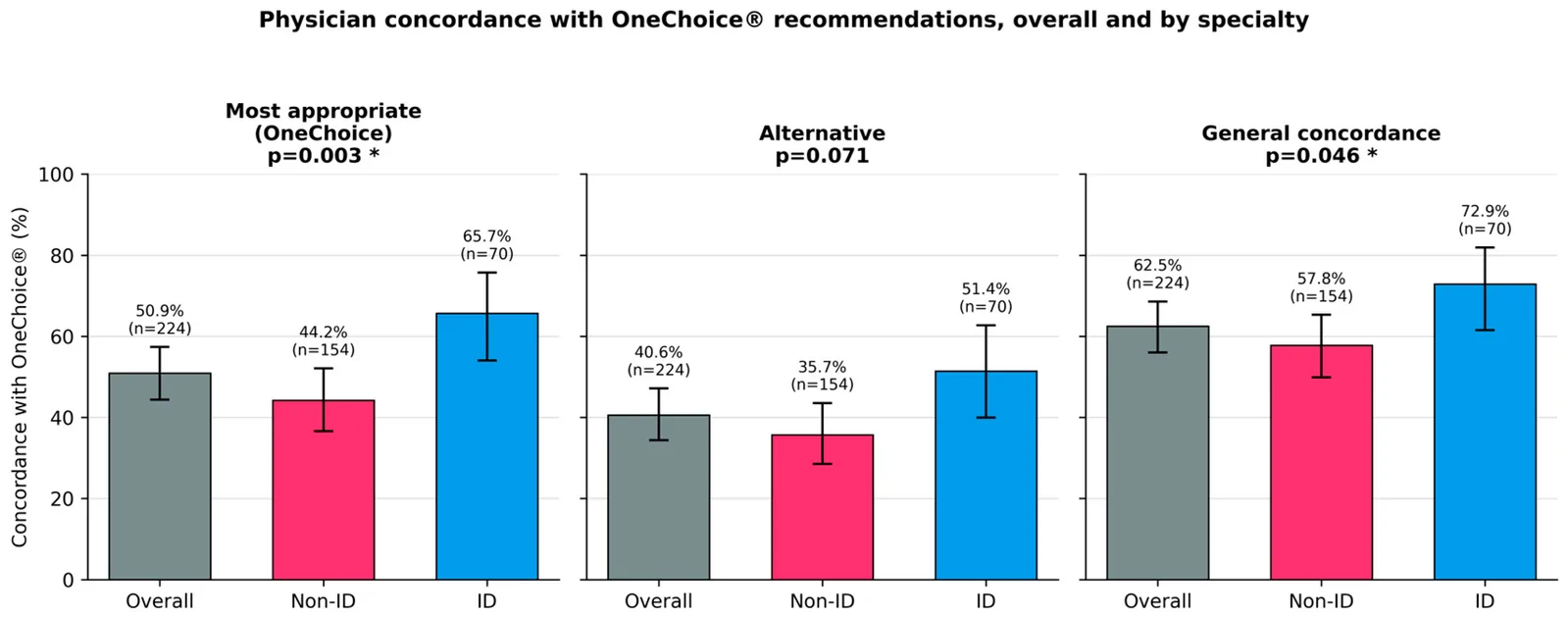
Concordance between physicians and OneChoice^®^, by outcome and specialty (ID vs. non-ID). Physician concordance with OneChoice^®^ recommendations, overall and by specialty. Concordance was evaluated for the primary recommendation, alternative recommendation, and overall concordance. Bars represent the proportion of physicians whose recommendations agreed with OneChoice^®^. Error bars indicate 95% confidence intervals. * *p*-values compare ID and non-ID physicians and were calculated using two-way cluster-robust standard errors at the physician and clinical-case levels.

Because the 224 evaluations were cross-classified by 194 physicians and 42 clinical cases, comparisons between specialty groups were conducted using two-way cluster-robust standard errors at the physician and clinical-case levels. Concordance was higher among ID specialists than among non-ID physicians for all three outcomes. After accounting for both sources of clustering, the difference was statistically significant for the primary recommendation (65.7% versus 44.2%; *p* = 0.003) and general concordance (72.9% versus 57.8%; *p* = 0.046), but not for the alternative recommendation (51.4% versus 35.7%; *p* = 0.071) (Table 2a,b and Figure 2).

In multivariable logistic regression models using two-way cluster-robust standard errors at the physician and clinical-case levels, ID specialty remained independently associated with concordance across all three outcomes: adjusted OR 2.62 (95% CI 1.45–4.73; *p* = 0.001) for the primary recommendation, 2.20 (95% CI 1.18–4.11; *p* = 0.013) for the alternative recommendation, and 2.14 (95% CI 1.11–4.14; *p* = 0.023) for general concordance. None of the other covariates included—physician age, years of specialty experience, UTI classification, or causative organism—were statistically significant in any of the three models (Table 3).

The other three models converged, with 22.8 outcome events per predictor for primary concordance, 18.2 for alternative concordance, and 28.0 for general concordance. Variance inflation factors ranged from 1.01 to 2.13, providing no evidence of problematic multicollinearity. Overall likelihood-ratio tests were significant for the primary (χ^2^ = 19.01, df = 5; *p* = 0.002), alternative (χ^2^ = 19.44, df = 5; *p* = 0.002), and general concordance models (χ^2^ = 16.37, df = 5; *p* = 0.006). The corresponding C-statistics were 0.663, 0.682, and 0.659, indicating modest discrimination. Hosmer–Lemeshow tests showed no evidence of lack of fit (*p* = 0.879, *p* = 0.160, and *p* = 0.990, respectively) (Supplementary S3).

To resolve discordant evaluations without relying on adjudication by the study co-authors, an independent, blinded panel of external experts—who had evaluated the same clinical vignettes against GPT-4 and Gemini in a separate study—served as the reference standard. Among the 110 discordant evaluations for the most appropriate recommendation, the external panel favored OneChoice^®^ in 102 (92.7%, 95% CI 86.3–96.3) and the physician in only 8 (7.3%). A similar pattern was observed for the alternative recommendation (124/133, 93.2%, 95% CI 87.6–96.4, favoring OneChoice^®^) and for general concordance (76/84, 90.5%, 95% CI 82.3–95.1). This pattern held across specialties: among ID specialists, OneChoice^®^ was favored in 91.7–94.1% of discordances, and among non-ID physicians in 90.8–93.0%, indicating that the algorithm’s advantage in discordant cases was not restricted to, or dependent on, the specialty of the disagreeing physician (Table 4a,b).

## 4. Discussion

In this study, the empirical antimicrobial recommendations issued by OneChoice^®^, an AI-powered, machine-learning-with-human-in-the-loop (ML-HITL) clinical decision support system (CDSS), showed only moderate agreement with participating physicians for the empirical management of urinary tract infections (UTIs), with overall concordance of 50.9% for the most appropriate recommendation, 40.6% for the alternative, and 62.5% for general concordance (agreement with at least one of the two options). Because some physicians completed more than one questionnaire and individual clinical cases were evaluated by multiple physicians, inferential comparisons and multivariable models used two-way cluster-robust standard errors at the physician and clinical-case levels. After accounting for both sources of dependence, concordance remained significantly higher among ID specialists for the primary recommendation and general concordance, whereas the unadjusted difference for the alternative recommendation was attenuated and was no longer statistically significant. In the multivariable models, however, ID specialty remained independently associated with all three concordance outcomes, with adjusted ORs ranging from 2.14 to 2.62. None of the other included physician- or case-level covariates reached statistical significance. Among discordant evaluations, the independent, blinded external panel judged the OneChoice^®^ recommendation as preferable in 92.7% of primary-recommendation discordances, 93.2% of alternative-recommendation discordances, and 90.5% of general-concordance cases. These findings indicate that, when physician and OneChoice^®^ recommendations differed, the OneChoice^®^ recommendation was frequently aligned with the external panel’s assessment. Because concordant evaluations were not independently adjudicated, these results should not be interpreted as demonstrating the overall superiority of OneChoice^®^ across all evaluations or clinical encounters. Moreover, expert adjudication serves as a reference assessment of treatment appropriateness rather than as evidence of improved clinical outcomes.

The magnitude and direction of this pattern are aligned with the previous performance of the same system in bacteremia, in which OneChoice^®^ reached 96.1% for any suggested treatment and 74.6% for the top recommendation, with significantly higher agreement among ID specialists (κ = 0.78) than among non-specialists (κ = 0.61) [24]. The present study extends this evidence in two relevant directions. First, it confirms the system’s behavior in a different clinical syndrome characterized by Enterobacterales predominance and a high prevalence of resistant phenotypes (ESBL-positive 37.1%, fluoroquinolone-resistant 47.3%, multidrug-resistant 39.3%). Second, it reinforces the methodological approach of resolving physician– OneChoice^®^ discordances through an external, blinded panel of experts rather than through adjudication by the study’s own co-authors, addressing a limitation that had been explicitly acknowledged in the bacteraemia study [24] and building on our recent head-to-head evaluation against general-purpose large language models, in which OneChoice^®^ achieved the highest agreement and sensitivity against an independent ID panel [25]. Taken together, these studies provide preliminary evidence that ML-HITL CDSS recommendations can show reproducible agreement with specialist or expert assessments across different infectious syndromes. Further prospective validation is required before concluding that this approach improves antimicrobial stewardship or patient outcomes.

The performance observed for OneChoice^®^ is also consistent with the broader AI landscape in stewardship. A recent systematic review of CDSS evaluating AI-guided antimicrobial therapy reported that ML-driven systems integrated into clinical workflows reduced extended-spectrum antibiotic days by 28.4% in pneumonia and 17.4% in UTIs, while general-purpose large language models showed higher prescribing error rates and patient safety risks [17]. A meta-analysis comparing AI tools with traditional risk-scoring systems demonstrated significantly higher sensitivity (pooled effect 1.93, *p* = 0.009) and negative predictive value (1.66, *p* < 0.001) for ML algorithms in stewardship-related outcomes [16]. Pathogen-specific ML models trained on MALDI-TOF spectra reached AUC values of 0.91–0.95 for predicting resistance in *K. pneumoniae* and *P. aeruginosa* [28], and electronic-health-record–based ML models identified ICU patients carrying multidrug-resistant pathogens within the first 24 h of admission with AUROC values of 0.786 (Random Forest) and 0.744 (XGBoost) [29]. In contrast to models primarily designed to predict antimicrobial resistance, the present study assessed concordance in antimicrobial regimen selection. Among discordant evaluations, the external panel more frequently judged the OneChoice^®^ recommendation to be preferable. This finding should be interpreted within the discordant subset and does not establish superiority across all physicians–case evaluations.

A second salient finding is the gap between ID specialists and non-specialists. ID physicians agreed with OneChoice^®^ for the most appropriate recommendation in 65.7% of evaluations, compared with 44.2% among non-specialists—a pattern that reproduces the bacteremia results [24], persists after adjustment for physician age and years of experience, and is consistent with telehealth-based stewardship interventions that increased prescribing appropriateness from 49.0% to 67.5% (aOR 2.48) and guideline adherence from 33.7% to 54.1% (aOR 2.44) when remote ID expertise was made available to non-specialist prescribers in rural settings [30]. These findings suggest that OneChoice^®^ may provide additional decision support to non-ID physicians in settings where direct ID consultation is limited. However, the present study did not establish that the system reproduces specialist-level reasoning or improves clinical outcomes, and these hypotheses require prospective evaluation.

The clinical implications are particularly relevant for UTIs. Selective reporting of antibiograms has been shown to reduce third-generation cephalosporin prescribing by 8.5% in outpatient UTI care [31], and bacterial resistance in *K. pneumoniae* has escalated globally—cephalosporin resistance from approximately 45% to 70% and carbapenem resistance from 25% to 50% over the last five years [32]—eroding the empirical reliability of widely used regimens. In this context, the external panel’s preference for OneChoice^®^ in 90.5–93.2% of discordant evaluations suggests that the system may provide useful decision support when its recommendation differs from that of the treating physician. Nevertheless, this adjudication-based finding does not establish clinical superiority or demonstrate that the use of CDSS improves patient outcomes.

This study has several strengths. First, discordant evaluations were resolved by an external, blinded panel of experts who had independently assessed the same clinical vignettes in a separate study, without the involvement of the present study’s co-authors—thereby minimizing the risk of adjudication bias that affects many OneChoice^®^ evaluations that rely on investigator judgment. Second, all comparisons and the multivariable model accounted for the non-independence of observations arising from physicians who completed more than one questionnaire, strengthening the validity of the inferential statistics. Third, the use of real, fully susceptibility-tested microbiological cases preserves external validity for ambulatory UTI care in Latin America. Fourth, the inclusion of three concordance levels (most appropriate, alternative, general) characterizes the system’s behavior beyond a single binary endpoint, which is methodologically aligned with the multi-tier structure of modern CDSS.

Several limitations warrant consideration. First, physicians were recruited using non-probabilistic convenience sampling through professional contacts, hospital networks, medical congresses, and electronic communication channels. Voluntary respondents may have had greater interest in infectious diseases, antimicrobial stewardship, or digital clinical tools than non-participants and may differ from the broader physician population in training, practice setting, and prescribing behavior. This selection mechanism could have influenced the observed concordance estimates and precludes interpreting them as population-representative estimates for physicians in Peru. Second, the study was conducted in a single country, and the participating physician sample is convenience-based, which may limit its generalizability to other healthcare systems. Although the QR-based randomization of cases mitigates ordering bias, the survey format does not capture longitudinal outcomes such as clinical cure, hospital readmission, or *Clostridioides difficile* infection; these outcomes will require prospective implementation studies. The number of participants in some specialty subgroups was small, and the prespecified multivariable model was restricted to a limited set of covariates to preserve an adequate events-per-variable ratio, potentially missing clinically relevant determinants of concordance. Finally, although the external expert panel was blinded to the source of each recommendation, it is not equivalent to a true clinical outcome; future work should pair the OneChoice^®^ recommendation with longitudinal patient outcomes to estimate causal effects on stewardship metrics and resistance trajectories.

## 5. Conclusions

In this cross-sectional concordance study, physicians agreed with the OneChoice^®^ primary recommendation in approximately half of the evaluations, with higher concordance among ID specialists than among non-ID physicians. When physician and OneChoice^®^ recommendations differed, an independent, blinded external panel judged the OneChoice^®^ recommendation to be preferable in most discordant evaluations. These results indicate frequent alignment between the OneChoice^®^ recommendation and expert assessment within the discordant subset, rather than overall clinical superiority of OneChoice^®^. Prospective implementation studies that incorporate clinical, microbiological, safety, and antimicrobial stewardship outcomes are required to determine the clinical utility of OneChoice^®^-assisted antimicrobial selection.

## Figures and Tables

**Table 1:** Case and physician characteristics, stratified by specialty (*N* = 224 evaluations).

Variable	Total (*n* = 224)	Non-ID (*n* = 154)	ID (*n* = 70)	*p*-Value
Total evaluations, *n*	224	154	70	
Years of specialty experience:				0.610 ^a^
0–5 years	85	55	30	
6–15 years	83	59	24	
16–20 years	16	13	3	
>20 years	40	27	13	
*Escherichia coli*	152	104	48	1.000 ^a^
*Klebsiella pneumoniae*	26	18	8	1.000 ^a^
*Proteus mirabilis*	16	15	1	0.050 ^b^
*Enterococcus faecalis*	9	5	4	0.466 ^b^
*Staphylococcus saprophyticus*	6	2	4	0.078 ^b^
*Pseudomonas aeruginosa*	6	3	3	0.380 ^b^
ESBL positive	83	55	28	0.641 ^a^
MDR status	88	59	29	0.768 ^a^
FQ resistance	106	71	35	0.691 ^a^
UTI type: Uncomplicated	198	136	62	1.000 ^a^
UTI type: Complicated	26	18	8	1.000 ^a^
Concordance (OneChoice vs. participant, primary)	114	68	46	0.004 ^a^

ESBL, extended-spectrum β-lactamase; FQ, fluoroquinolone; ID, infectious disease specialist; MDR, multidrug-resistant; UTI, urinary tract infection. ^a^ Pearson’s χ^2^ test or ^b^ Fisher’s exact test.

**Table 2:** (**a**) Concordance between physicians and OneChoice^®^ recommendations, by outcome and specialty group (Wilson 95% CI). (**b**) Concordance comparison between ID and non-ID specialists using two-way cluster-robust standard errors.

**(a)**					
**Group**	**Outcome**	***n***	**Agreements**	**Agreement %**	**95% CI**
All participants	Primary (OneChoice)	224	114	50.9%	44.4–57.4
All participants	Alternative	224	91	40.6%	34.4–47.2
All participants	General concordance	224	140	62.5%	56.0–68.6
ID specialists	Primary (OneChoice)	70	46	65.7%	54.0–75.8
ID specialists	Alternative	70	36	51.4%	40.0–62.8
ID specialists	General concordance	70	51	72.9%	61.5–81.9
Non-ID specialists	Primary (OneChoice)	154	68	44.2%	36.5–52.0
Non-ID specialists	Alternative	154	55	35.7%	28.6–43.5
Non-ID specialists	General concordance	154	89	57.8%	49.9–65.3
**(b)**					
**Outcome**		**ID Specialists**		**Non-ID Specialists**	***p*-Value (Cluster-Robust)**
Primary (OneChoice)		65.7%		44.2%	0.003
Alternative		51.4%		35.7%	0.071
General concordance		72.9%		57.8%	0.046

CI, confidence interval; ID, infectious disease specialist; non-ID, non-infectious disease specialist. Data are presented as *n* and percentages. The 95% confidence intervals were calculated using the Wilson score method. *p*-values were calculated using two-way cluster-robust standard errors, with clustering at the physician and clinical-case levels and finite-sample corrections.

**Table 3:** Multivariable logistic regression for concordance with OneChoice^®^, using two-way cluster-robust standard errors at the physician and clinical-case levels (*N* = 224).

Covariate	Primary-aOR (95% CI)	*p*-Value	Alternative-aOR (95% CI)	*p*-Value	General-aOR (95% CI)	*p*-Value
ID specialist (vs. non-ID)	2.68 (1.45–4.94)	0.002	2.23 (1.23–4.04)	0.008	2.19 (1.16–4.17)	0.016
Physician age (per year)	1.02 (0.98–1.06)	0.328	1.03 (0.99–1.07)	0.143	1.03 (1.00–1.07)	0.079
Years of specialty experience (per year)	1.01 (0.97–1.04)	0.628	1.04 (0.98–1.09)	0.171	1.02 (0.97–1.07)	0.502
UTI complicated (vs. uncomplicated)	0.54 (0.22–1.35)	0.188	0.91 (0.37–2.26)	0.859	0.96 (0.39–2.34)	0.930
*E. coli* (vs. other pathogens)	1.75 (0.94–3.24)	0.079	1.11 (0.60–2.04)	0.738	1.73 (0.92–3.26)	0.089

aOR, adjusted odds ratio; CI, confidence interval; ID, infectious disease specialist; non-ID, non-infectious disease specialist; UTI, urinary tract infection. Odds ratios > 1 indicate greater concordance with OneChoice^®^. The models included the prespecified covariates shown in the table. Years of specialty experience were represented using the midpoint of each recorded category. Confidence intervals and *p*-values were estimated using two-way cluster-robust standard errors at the physician and clinical-case levels, with finite-sample corrections.

**Table 4:** (**a**) Resolution of discordant evaluations by the independent external expert panel (Wilson 95% CI). (**b**) Resolution of discordant evaluations by the external expert panel, stratified by specialist group.

**(a)**				
**Outcome**	**Total Discordances**	**Favors OneChoice, *n* (%)**	**95% CI**	**Favors Participant, *n* (%)**
Primary (OneChoice)	110	102 (92.7%)	86.3–96.3	8 (7.3%)
Alternative	133	124 (93.2%)	87.6–96.4	9 (6.8%)
General concordance	84	76 (90.5%)	82.3–95.1	8 (9.5%)
**(b)**				
**Specialist Group**	**Outcome**	**Total Discordances**	**Favors OneChoice, *n* (%)**	**Favors Participant, *n* (%)**
ID	Primary (OneChoice)	24	22 (91.7%)	2 (8.3%)
ID	Alternative	34	32 (94.1%)	2 (5.9%)
ID	General concordance	19	17 (89.5%)	2 (10.5%)
Non-ID	Primary (OneChoice)	86	80 (93.0%)	6 (7.0%)
Non-ID	Alternative	99	92 (92.9%)	7 (7.1%)
Non-ID	General concordance	65	59 (90.8%)	6 (9.2%)

CI, confidence interval. Data are presented as *n* (%). The 95% confidence intervals were estimated using the Wilson score method. ID, infectious disease specialist; non-ID, non-infectious disease specialist.

## Data Availability

The data presented in this study are openly available at https://doi.org/10.6084/m9.figshare.32924888.

## Supplementary Materials

The following supporting information can be downloaded at: https://www.mdpi.com/article/10.3390/diagnostics16172708/s1, Supplementary S1: STROBE Statement—Checklist; Supplementary S2: Questionnaire selection; Supplementary S3. Diagnostic performance and goodness-of-fit measures for the multivariable logistic regression models.
