# Supplementary material for: Comparison of OneChoice AI-Based Clinical Decision Support Recommendations with Infectious Disease Specialists and Non-Specialists for Empirical Urinary Tract Infection Therapy in Lima, Peru
Source: Diagnostics (Basel). 2026 Aug 25;16(17):2708. doi: 10.3390/diagnostics16172708 (PMC13565701; doi:10.3390/diagnostics16172708)
Supplement: Supplementary file 1 [file diagnostics-16-02708-s001.zip › diagnostics-4456951-supplementary.pdf]

Supplementary S1: STROBE Statement—Checklist

Manuscript: *Comparison of OneChoice AI-based clinical decision support recommendations with infectious disease specialists and non-specialists for empirical urinary tract infection therapy in Lima, Peru*

Reporting guideline: STROBE checklist for cross-sectional studies (version 4).

Section/topic	Item no.	STROBE recommendation	Reported on page no.
**Title and abstract**	**1(a)**	Indicate the study's design with a commonly used term in the title or the abstract.	Page 3 (Abstract: "cross-sectional, survey-based concordance study").
	**1(b)**	Provide in the abstract an informative and balanced summary of what was done and what was found.	Page 3 (Abstract).
**Introduction—Background/rationale**	**2**	Explain the scientific background and rationale for the investigation being reported.	Pages 4–6 (Introduction).
**Introduction—Objectives**	**3**	State specific objectives, including any prespecified hypotheses.	Page 6 (final paragraph of the Introduction).
**Methods—Study design**	**4**	Present key elements of study design early in the paper.	Page 7 (Section 2.1, Study design and setting).
**Methods—Setting**	**5**	Describe the setting, locations, and relevant dates, including periods of recruitment, exposure, follow-up, and data collection.	Page 7 (Section 2.1: Lima, Peru; source-case and survey periods).

Section/topic	Item no.	STROBE recommendation	Reported on page no.
**Methods—Participants**	**6(a)**	Give the eligibility criteria, and the sources and methods of selection of participants.	Pages 8–9 (Section 2.3, Participant recruitment and eligibility criteria).
**Methods—Variables**	**7**	Clearly define all outcomes, exposures, predictors, potential confounders, and effect modifiers. Give diagnostic criteria, if applicable.	Pages 10–12 (Section 2.6, Data preparation, outcome definitions, and data analysis).
**Methods—Data sources/measurement**	**8***	For each variable of interest, give sources of data and details of methods of assessment (measurement). Describe comparability of assessment methods if there is more than one group.	Pages 7–10 (Sections 2.2–2.4 and 2.6: source records, clinical questionnaires, OneChoice® recommendations, physician survey responses, and concordance definitions).
**Methods—Bias**	**9**	Describe any efforts to address potential sources of bias.	Pages 7–9 (case completeness criteria, randomized questionnaire assignment, and participant criteria); pages 20–21 (external blinded adjudication); page 25 (limitations and potential selection/conflict-of-interest biases).

Section/topic	Item no.	STROBE recommendation	Reported on page no.
**Methods—Study size**	**10**	Explain how the study size was arrived at.	Pages 8–9 and 13–14 (81 candidate vignettes screened, 42 included, and 224 physician–case evaluations from 194 physicians). No a priori sample-size calculation was reported.
**Methods—Quantitative variables**	**11**	Explain how quantitative variables were handled in the analyses. If applicable, describe which groupings were chosen and why.	Page 12 (years of specialty experience collected in ordered ranges and represented by category midpoints as a continuous variable).
**Methods—Statistical methods**	**12(a)**	Describe all statistical methods, including those used to control for confounding.	Pages 11–12 (descriptive comparisons, Wilson confidence intervals, multivariable logistic regression, prespecified covariates, and two-way cluster-robust covariance estimation at physician and clinical-case levels with finite-sample corrections).
	**12(b)**	Describe any methods used to examine subgroups and interactions.	Pages 11–12 (subgroup analyses stratified by physician and clinical-case characteristics). No interaction analysis was reported.

Section/topic	Item no.	STROBE recommendation	Reported on page no.
	**12(c)**	Explain how missing data were addressed.	Pages 10–11 (no missing outcome or model-covariate data; no imputation; complete-case analysis included all 224 evaluations).
	**12(d)**	If applicable, describe analytical methods taking account of sampling strategy.	Not applicable: physicians were recruited by convenience, questionnaires were randomly assigned, and no probability-sampling weights were used. The cross-classified dependence was addressed with two-way cluster-robust covariance estimation (pages 9 and 11–12).
	**12(e)**	Describe any sensitivity analyses.	Not applicable: no sensitivity analysis was reported. Model diagnostics were reported on page 12.
**Results—Participants**	**13(a)**	Report numbers of individuals at each stage of study—e.g., numbers potentially eligible, examined for eligibility, confirmed eligible, included in the study, completing follow-up, and analyzed.	Pages 13–14 (Results and Figure 1: 81 candidate vignettes, 42 included vignettes, 194 unique physicians, and 224 physician–case evaluations).

Section/topic	Item no.	STROBE recommendation	Reported on page no.
	**13(b)**	Give reasons for non-participation at each stage.	Pages 7–8 and 13–14 (case-vignette exclusion criteria and study flow); page 9 (physician exclusion criteria).
	**13(c)**	Consider use of a flow diagram.	Pages 13–14 (Figure 1, Study flow diagram).
**Results—Descriptive data**	**14(a)***	Give characteristics of study participants (e.g., demographic, clinical, social) and information on exposures and potential confounders.	Pages 14–16 (Table 1: physician and clinical-case characteristics, overall and by specialty group).
	**14(b)***	Indicate number of participants with missing data for each variable of interest.	Pages 10–11 (no missing values for the three outcomes or covariates included in the multivariable models).
**Results—Outcome data**	**15***	Report numbers of outcome events or summary measures.	Pages 16–18 (Tables 2a and 2b: concordance counts, proportions, and confidence intervals).
**Results—Main results**	**16(a)**	Give unadjusted estimates and, if applicable, confounder-adjusted estimates and their precision (e.g., 95% confidence interval). Make clear which confounders were adjusted for and why they were included.	Pages 16–19 (Tables 2a–2b and 3: unadjusted concordance estimates and adjusted odds ratios with 95% confidence intervals; covariates specified in Section 2.6 on page 12).

Section/topic	Item no.	STROBE recommendation	Reported on page no.
	**16(b)**	Report category boundaries when continuous variables were categorized.	Pages 14–15 (Table 1: physician age and specialty-experience categories); page 12 (handling of specialty-experience categories using category midpoints).
	**16(c)**	If relevant, consider translating estimates of relative risk into absolute risk for a meaningful time period.	Not applicable: the outcomes were cross-sectional concordance measures, not time-dependent risks.
**Results—Other analyses**	**17**	Report other analyses done—e.g., analyses of subgroups and interactions, and sensitivity analyses.	Pages 19–21 (model diagnostics and external-panel adjudication of discordant evaluations; Tables 4a and 4b). No sensitivity or interaction analyses were reported.
**Discussion—Key results**	**18**	Summarize key results with reference to study objectives.	Pages 21–22 (opening paragraphs of the Discussion).
**Discussion—Limitations**	**19**	Discuss limitations of the study, considering sources of potential bias or imprecision. Discuss both direction and magnitude of any potential bias.	Pages 24–25 (Discussion, limitations).

**Section/topic**	**Item no.**	**STROBE recommendation**	**Reported on page no.**
**Discussion—Interpretation**	**20**	Give a cautious overall interpretation of results considering objectives, limitations, multiplicity of analyses, results from similar studies, and other relevant evidence.	Pages 21–26 (Discussion and Conclusions).
**Discussion—Generalizability**	**21**	Discuss the generalizability (external validity) of the study results.	Page 25 (limitations concerning convenience sampling, the Peruvian healthcare context, local resistance patterns, and clinical practice).
**Other information—Funding**	**22**	Give the source of funding and the role of the funders for the present study and, if applicable, for the original study on which the present article is based.	Page 27 (Funding and Conflicts of Interest statements).

* Give information separately for exposed and unexposed groups, as applicable.

STROBE source: STROBE Statement—Checklist of items that should be included in reports of cross-sectional studies, version 4: https://www.strobe-statement.org/fileadmin/Strobe/uploads/checklists/STROBE_checklist_v4_cross-sectional.pdf

Supplementary S2: Questionnaire selection

Correlation between the clinical decision-based system (Onechoice®) with recommendations from physicians of various specialties for the choice of antimicrobials in urinary tract infections

Greetings, colleague. Thank you very much for participating in this study. This analysis compares pharmacotherapy decision-making based on healthcare professionals' experience with alternatives provided by a Human-in-the-Loop Machine Learning system developed by Arkstone Medical Solution. This survey is aimed at healthcare professionals with a medical specialty, so no additional personal data beyond what is asked for is necessary.

The goal is to select the best therapeutic option, including dosage and duration of the selected antimicrobial, based on the results provided. Please consider yourself in a scenario where only the pharmacotherapy available in your country can be employed. Some cases include conditional events like recent antibiotic use, allergies, and other particular conditions. This information will appear in the question. Avoid using guidelines or virtual tools that might assist you in your decision-making process.

Iniciar sesión en Google para guardar lo que llevas hecho. Más información

* Indica que la pregunta es obligatoria

To start the survey, please read the informed consent and select the ACCEPT option to continue if you accept them - https://docs.google.com/document/d/1rQPgiQU7_HwCCh68yqHO-KhGtoA1mL4To47mraPvcQ0/edit?usp=sharing : *

- ○ Accept
- ○ Reject

Siguiente

Borrar formulario

CASE 090 (AO_7729_302086839): The patient is a 79-year-old female with hypertension and congestive Heart Failure. She comes to a clinical appointment with a 4-day history of cloudy urine and mild dysuria. No fever and tolerates oral intake. A urinalysis was performed, which showed abnormal results. The urine culture report is attached. Which of the following antimicrobial options would you recommend? Please consider dosage, interval, frequency, and duration of treatment. The patient does not have any allergies. **Your recommendation should align with the Principles of Antimicrobial stewardship and current guidelines.**

* 5 puntos

Klebsiella pneumoniae ✓

SENSITIVITIES

Amikacin	S
Amox/Clav	S
AMP/SUL	S
Aztreonam	S
Cefazolin	S
Cefepime	S
Cefixime	S
Cef/Sul	S
Cefotaxime	S
Ceftazidime	S
Ceftriaxone	S
Cefuroxime	S
Cipro	S
Ertapenem	S
Fosfomycin	S
Gentamicin	S
Levofloxacin	S
Meropenem	S
Nalidixic acid	S
Macrobid	S
Pip/Taz	S
TMP-SMX	S

- Nitrofurantoin XR 100 mg PO BID x 5 days
- Cefixime 400 mg PO every 24h x 5-7 days
- Amoxicillin/Clavulanic acid 500/125 mg PO every 12h x 5-7 days
- Fosfomycin 3 g PO for 1 dose
- Trimethoprim-sulfamethoxazole DS 1 tablet PO BID x 3 days

If your first recommended option is unavailable, which antimicrobial options would you recommend as an alternative?

* 5 puntos

- Amoxicillin/Clavulanic acid 500/125 mg PO every 12h x 5-7 days
- Nitrofurantoin XR 100 mg PO BID x 5 days
- Fosfomycin 3 g PO for 1 dose
- Cefixime 400 mg PO every 24h x 5-7 days
- Trimethoprim-sulfamethoxazole DS 1 tablet PO BID x 3 days

Atrás | Enviar | Borrar formulario

Supplementary S3. Diagnostic performance and goodness-of-fit measures for the multivariable logistic regression models.

Diagnostic	Primary	Alternative	General
**N**	224	224	224
**Events**	114	91	140
**Events per predictor**	22.8	18.2	28.0
**Maximum VIF**	2.13	2.13	2.13
**Likelihood-ratio χ^2**	19.01	19.44	16.37
**LR-test p-value**	0.002	0.002	0.006
**C-statistic**	0.663	0.682	0.659
**Brier score**	0.230	0.221	0.218
**Hosmer–Lemeshow p-value**	0.879	0.160	0.990
